# Chitosan Nanoparticles Combined With Titanium-Prepared Platelet-Rich Fibrin for Socket Preservation in Postextraction Sites: Protocol for a Single-Arm Clinical, Radiographic, and Histomorphometric Study

**DOI:** 10.2196/79532

**Published:** 2026-10-06

**Authors:** Rutuja Karamore, Prasad Dhadse, Sanehi Punse

**Affiliations:** 1 Department of Periodontology and Implantology Sharad Pawar Dental College Datta Meghe Institute of Higher Education and Research Wardha, Maharashtra India

**Keywords:** bone regeneration, chitosan, histomorphometry, socket preservation, titanium-prepared platelet-rich fibrin

## Abstract

**Background:**

Alveolar ridge resorption following tooth extraction can compromise subsequent implant placement. Socket preservation techniques aim to minimize dimensional changes and promote favorable bone healing. Chitosan nanoparticles combined with titanium-prepared platelet-rich fibrin represent a biologically active approach to ridge preservation.

**Objective:**

This study aims to evaluate longitudinal changes in alveolar ridge dimensions and bone quality following socket preservation using chitosan nanoparticles combined with titanium-prepared platelet-rich fibrin.

**Methods:**

This is a single-arm prospective clinical study involving systemically healthy participants requiring tooth extraction. Clinical, radiographic, and histomorphometric assessments will be performed at baseline and at follow-up visits at 3 and 6 months. Statistical analysis will be conducted using within-subject repeated-measures approaches.

**Results:**

No external funding was received for this research. The manuscript was submitted before recruitment of participants had started and no clinical data had been collected or analyzed. Clinical, radiographic, and histomorphometric outcomes will be evaluated to assess changes in the dimensions of the alveolar ridge, bone density, and quality of regenerated bone during the postextraction healing period. Clinical and radiographic evaluation will be performed at baseline, 3 months, and 6 months. Histomorphometric analysis will be performed on bone core biopsy samples taken at the time of implant placement. The results of the study will be reported after data collection and analysis.

**Conclusions:**

This protocol outlines a standardized approach to evaluating the clinical, radiographic, and histomorphometric outcomes of socket preservation using chitosan nanoparticles combined with titanium-prepared platelet-rich fibrin. The findings from this study may inform the design of future controlled clinical trials.

**Trial Registration:**

Clinical Trials Registry of India CTRI/2025/06/089715; https://tinyurl.com/4u6698xs

**International Registered Report Identifier (IRRID):**

PRR1-10.2196/79532

## Introduction

Ridge preservation is a crucial procedure in dentistry that aims to maintain the alveolar bone structure and integrity following tooth extraction. Most cases that require bone restoration are caused by osteolysis associated with periodontal disorders. The loss of alveolar bone after tooth extraction can lead to functional and esthetic complications, making ridge preservation an essential step for successful prosthetic or implant rehabilitation [1].

Applications for chitosan and its derivatives in biomedicine are numerous. Because chitosan, a biopolymer made from chitin, is biocompatible, biodegradable, antibacterial, and can encourage cell adhesion and proliferation, it has drawn a lot of interest in bone regeneration [2,3]. When combined with nanoparticles, such as hydroxyapatite, β-tricalcium phosphate (TCP), or bioactive glass, chitosan-based scaffolds offer enhanced osteoconductive and osteoinductive properties [4,5].

Chitosan nanoparticles (CNPs) have an ordered morphology and good cytocompatibility. After extraction, their broad-spectrum antibacterial activity is essential for preserving a sterile healing environment. These nanoparticles work well against a variety of oral pathogens that are frequently linked to peri-implant and periodontal diseases. Among these pathogens, CNPs exhibit a high inhibitory effect on *Porphyromonas gingivalis*, a significant periodontal pathogen. Additionally, nanoparticles successfully reduced the gene and protein expression of the nucleotide-binding oligomerization domain–like receptor protein 3 (NLRP3) inflammasome and interleukin-1β (IL-1β) in human gingival fibroblasts (HGFs). Furthermore, they accelerate the bone-formation process of bone marrow mesenchymal stromal cells (BMSCs), thereby increasing the expression of genes associated with bone development such as bone sialoprotein (BSP), osteocalcin, collagen type I (Col I), and osteopontin. Furthermore, CNPs have been shown to improve the mineralization of the extracellular matrix [6].

CNPs aid in ridge preservation through multiple mechanisms. They serve as scaffolds for osteoblast proliferation and new bone formation, promoting effective bone regeneration [6]. Their biodegradable nature ensures gradual degradation into nontoxic by-products while being replaced by natural bone [7]. Additionally, chitosan exhibits inherent antimicrobial properties, helping to prevent infections and reduce postextraction complications [8,9]. Moreover, its porous structure allows for controlled drug delivery, enabling the incorporation of growth factors or antibiotics that further enhance bone healing [10].

CNPs offer several clinical applications and advantages in ridge preservation. They help maintain bone volume by preserving alveolar dimensions, which are crucial for successful implant placement [4]. By reducing bone resorption, these biomaterials slow down the natural bone loss that typically occurs after tooth extraction [5]. Their bioactive properties contribute to faster healing of both soft and hard tissues, promoting overall recovery [2]. Additionally, their effectiveness in preserving bone structure reduces the need for additional grafting procedures, making them a cost-effective and efficient alternative for dental rehabilitation [3]. These combined properties provide a biological rationale for the use of CNPs in ridge preservation.

Given their biostimulatory potential, combining CNPs with autologous biologics such as titanium-prepared platelet-rich fibrin (T-PRF) may provide synergistic benefits. T-PRF offers a dense fibrin network, growth factors, and enhanced biocompatibility without chemical additives, supporting its use as an autologous biological adjunct in ridge preservation procedures.

Therefore, this study aims to evaluate longitudinal changes in alveolar ridge dimensions and bone quality following socket preservation using CNPs combined with T-PRF. Clinical and radiographic outcomes will be assessed at baseline, 3 months, and 6 months to correspond with established phases of postextraction bone remodeling, while histomorphometric outcomes will be evaluated at the time of implant placement.

## Methods

### Study Design

This study is designed as a single-arm, prospective clinical, radiographic, and histomorphometric protocol. All outcomes will be evaluated using within-subject comparisons across predefined follow-up time points.

### Ethical Considerations

The study protocol has been examined and approved by the Sharad Pawar Dental College, Datta Meghe Institute of Higher Education and Research institutional ethics committee (IEC; DMIHER (DU)/IEC/2025/752), ensuring that ethical standards and guidelines are followed. This statement confirms that informed consent, participant safety, and confidentiality will be maintained throughout the conduct of the study.

### Trial Registration

The Clinical Trials Registry of India has registered this single-arm trial under reference number REF/2025/05106568. This registration guarantees transparency, accountability, and adherence to ethical principles throughout the study.

### Sample Size Calculation

The sample size calculation was based on the primary outcome of the change in alveolar ridge width between baseline and the 6-month follow-up using paired mean differences.

Formulas used are as follows:

n ≥ (2 [Z(1–α/2) + Z(1–β)]^2^ [σ_Difference_/δ_Difference_]^2^) + (Z[1–α/2]^2^/2)

The given values are as follows:

Z(1–α/2) = 1.96 (for α=.05)= 1.96

Z(1–β) = 1.645 (for β=.05)

Mean difference (δ_Diff_) = 1.09

SD of the difference (σ_Diff_) = 0.82

n = (2 (1.96 + 1.645)^2^ (0.82/1.09)^2^) + (1.96^2^/2)

n = (2 (3.605)^2^ (0.752)) + (3.8416/2)

n = (2 (13.002) (0.752)) + 1.9208

n=(19.538) + 1.9208

n = 17

Thus, the sample size was determined to be 17.

The sample size calculation was based on the primary outcome variable of horizontal alveolar ridge width change, comparing baseline measurements with the 6-month follow-up. A paired-difference approach was applied, assuming a moderate correlation between repeated measurements over time. To account for potential participant attrition or incomplete follow-up, an allowance for dropout was incorporated into the final sample size estimation.

### Outcome Measures

The primary outcome is the change in alveolar ridge width from baseline to 3 and 6 months. Secondary outcomes include changes in ridge height and radiographic bone density, as well as histomorphometric parameters evaluated at the time of implant placement.

### Inclusion Criteria

Patients between the ages of 18 and 55 years who are in good overall health and do not smoke will be included in the trial. Participants must be eligible if they have teeth that need to be extracted because of residual roots, internal or external resorption, failed endodontic treatment, root fractures, or nonrestorable carious diseases. We will also take into account cases with severely decaying teeth or sections of the root without purulent discharge or cellulitis. It is also necessary for nearby teeth and an opposing natural tooth to be present. For the crestal main incision to close completely, sufficient soft tissue health is necessary.

### Exclusion Criteria

Participants with systemic health conditions, such as diabetes mellitus, osteoporosis, blood disorders, or a known titanium allergy, that may impede bone healing and regeneration will not be allowed to participate in the study. Patients with a history of drug abuse, heavy smoking, or alcohol consumption will also be excluded. Other exclusion criteria include pregnancy or lactation and the presence of untreated dental conditions.

Strict inclusion and exclusion criteria were applied to minimize the influence of potential confounding factors, such as systemic disease, smoking status, and impaired wound healing.

### Procedure

#### Initial Therapy

Nonsurgical periodontal therapy will be done 1 week before surgery. Clinical photographs will be taken throughout the process for all patients.

#### Clinical Parameters

The height of the ridge will be measured from the cementoenamel junction (CEJ) of the adjacent teeth to the occlusal surface of the adjacent teeth. The width of the ridge will be measured buccolingually using a UNC-15 calibrated periodontal probe (University of North Carolina, Hu-Friedy). It will be evaluated at baseline, 3 months, and 6 months. All clinical measurements will be performed using standardized reference points and calibrated periodontal probes to ensure measurement consistency across time points.

#### Radiographic Measurements

Cone beam computed tomography (CBCT) images will be taken on the day of surgery and at 3 months and 6 months after surgery to evaluate changes in alveolar ridge height, width, and bone density following tooth extraction.

On the basis of previous research conducted by Yosouf et al [11], a reference line approach was adopted to guarantee uniformity and prevent modifications brought on by patient placement. The axial and sagittal views revealed 2 fixed reference lines that went through the pulp canals and cementoenamel connections of the nearby teeth, respectively. To quantify alveolar height, width, and density using the third view window, these reference lines were set up in the coronal view. Fixed reference lines will be used in all CBCT measurements to minimize variability related to patient positioning and image orientation.

#### Histomorphometric Analysis

Using a trephine bur in a sterile environment, bone sample biopsies will be taken from the preserved socket after implant insertion. For histological analysis, these samples will be sectioned, decalcified, embedded in paraffin, and preserved in 10% buffered formalin. To assess the quality and morphology of the bone, sections will be stained with hematoxylin and eosin (H&E). Histomorphometric evaluation will be performed using predefined qualitative and semiquantitative scoring criteria. The examiner performing the histological assessment will be blinded to the clinical and radiographic outcomes.

The following histomorphometric parameters will be examined:

- New bone formation: the percentage of newly produced bone within the entire biopsy region
- Residual biomaterial: the percentage of chitosan–T-PRF complex material that is still present in the section
- Connective tissue area: the percentage of soft or fibrous tissue found in the biopsy
- Osteoid presence and maturity: evaluation of osteoid seams and the degree of bone maturation using qualitative and semiquantitative methods
- Vascularization: the assessment of recently developed blood vessels as a sign of tissue repair and integration
- Inflammatory cell infiltrate: the quantity and presence of inflammatory cells, evaluating the biocompatibility and the healing response

### Surgical Procedure

A preprocedural 0.2% chlorhexidinegluconate solution will be administered to patients for 1 minute before surgery. The procedure will be performed under all aseptic conditions and with all necessary precautions. A nerve block and infiltration with 2% Lignocaine at a 1:80,000 epinephrine concentration (Ligno-Ad local anesthetic; Proxim Remedies) will be used to anesthetize the area. A number 15 blade will be used for the incision. The periosteal elevator will be used to lift the full-thickness mucoperiosteal flap. Atraumatic extraction of the tooth and debridement of the socket will be done. After hemostasis is achieved, betadine irrigation will be done. The material will be placed into the socket, ensuring full coverage without excessive compression using an instrument. The surgical site will be sutured.

### Preparation of the Biomaterial

Preparation of T-PRF is done as follows:

- Collect venous blood from the patient into titanium-coated tubes.
- Centrifuge the tube at 2700 rpm for 13 minutes to separate T-PRF.
- Extract the fibrin clot and remove excess red blood cells and serum.
- Allow the T-PRF clot to polymerize into a bioactive membrane or use it in liquid form for mixing.

The procedure of coating the T-PRF membrane with CNP is as follows:

- Prepare the T-PRF membrane after clot formation.
- Suspend the CNPs in a small volume of sterile saline or platelet-poor plasma (PPP).
- Dip the T-PRF membrane into the CNP suspension for a few minutes to allow nanoparticle adhesion.
- Allow the membrane to dry slightly before placing it into the extraction site.

### Postoperative Care

After the treatment, systemic antibiotics will be prescribed to reduce postoperative pain and inflammation. Patients will be instructed not to brush their teeth in the treated area for 3 weeks following the treatment. At the end of each day, each patient will be instructed to rinse twice with 0.2% chlorhexidine gluconate (Hexidine; ICPA Health Products Ltd). Patients are called back for suture removal 1 week later. After 3 and 6 months following the procedure, the patients will be summoned for follow-ups.

Clinical and radiographic parameters will be evaluated at baseline, 3 months, and 6 months. Histomorphometric analysis will be done at the time of implant placement at 3 months.

Any postoperative complications or adverse events will be recorded and managed according to standard clinical protocols throughout the follow-up period.

### Statistical Analysis

Statistical analysis will be performed using standard statistical software. Continuous variables will be summarized as mean (SD). The normality of the data distribution will be assessed using the Shapiro-Wilk test.

For outcomes measured at multiple time points (baseline, 3 months, and 6 months), repeated-measures ANOVA will be used for normally distributed data. When a significant overall effect is observed, Bonferroni-adjusted post hoc tests will be applied for pairwise comparisons between time points.

If the assumption of normality is violated, the Friedman test will be used, followed by appropriate post hoc analyses.

Histomorphometric outcomes assessed at a single time point will be analyzed descriptively. Where applicable, exploratory paired comparisons with baseline radiographic outcomes may be performed.

Effect sizes and 95% CIs will be reported alongside *P* values. Missing data will be handled using complete-case analysis, as loss to follow-up is expected to be minimal. A 2-tailed *P* value <.05 will be considered statistically significant.

## Results

This research received no external funding. At the time of manuscript submission, participant recruitment had not begun and no clinical data had been collected or analyzed.

### Clinical Outcomes

Clinical measurements of alveolar ridge height and width will be recorded at baseline, and at 3 months and 6 months following socket preservation using CNPs combined with T-PRF. Measurements will be obtained using standardized reference points to ensure consistency across follow-up visits.

### Radiographic Outcomes

Radiographic assessment will be performed using CBCT to evaluate changes in alveolar ridge dimensions and bone density at baseline, 3 months, and 6 months. Measurements will be carried out using predefined reference lines and calibrated imaging parameters to maintain reproducibility.

### Histomorphometric Outcomes

Histomorphometric evaluation will be conducted on bone core biopsy samples obtained at the time of implant placement. Parameters including new bone formation, residual biomaterial, connective tissue components, vascularization, and inflammatory cell infiltration will be assessed using standardized scoring criteria.

## Discussion

This protocol outlines a single-arm, within-subject longitudinal study designed to evaluate clinical, radiographic, and histomorphometric changes following alveolar ridge preservation using CNPs combined with T-PRF.

### Principal Findings

This study is designed to generate preliminary clinical, radiographic, and histomorphometric data on alveolar ridge preservation using CNPs combined with T-PRF. By using a within-subject longitudinal design, the protocol facilitates the evaluation of dimensional changes, bone density variation, and qualitative characteristics of regenerated bone during the postextraction healing period. The integration of histomorphometric analysis provides a direct microscopic assessment of bone regeneration and biomaterial biocompatibility, complementing clinical and radiographic observations.

The biological properties of CNPs, including osteoconductivity, biodegradability, and antimicrobial activity, combined with the growth factor–rich fibrin matrix of T-PRF, provide a biological rationale for their combined use in alveolar ridge preservation. The integration of histomorphometric evaluation enables a direct microscopic assessment of bone regeneration and biomaterial biocompatibility. These properties are supported by prior evidence demonstrating that chitosan-based biomaterials function as biocompatible, biodegradable matrices that promote osteoblast adhesion, mineralization, and osteogenesis in bone regenerative applications.

Recent clinical trials have also shown that chitosan-containing scaffolds significantly reduce dimensional ridge loss and improve radiographic bone density following tooth extraction. For example, Al-Madhagy et al [12] reported reduced vertical and horizontal bone loss and higher mean bone density with chitosan or polyvinyl alcohol (PVA) nanofibrous matrices. On the basis of these findings, the current protocol is designed to further investigate the clinical, radiographic, and histomorphometric performance of CNPs in combination with autologous platelet concentrates for ridge preservation.

This protocol incorporates several methodological strengths, including the use of standardized CBCT measurements with fixed reference lines, which enhance the reliability and reproducibility of radiographic assessments. Histomorphometric analysis of bone samples obtained at implant placement will allow a direct microscopic evaluation of bone quality, residual biomaterial, and inflammatory response, providing biological validation of radiographic findings. The use of autologous biologics, such as T-PRF, offers improved biocompatibility and fibrin integrity without chemical additives, supporting a favorable safety profile. In addition, the incorporation of CNPs may enhance osteoconductivity and provide antimicrobial effects, potentially contributing to improved healing outcomes and reduced postoperative complications.

These design features are intended to support internal validity and enable a comprehensive evaluation of the intervention within a single-arm study framework.

A comprehensive review discusses the role of chitosan-based scaffolds in bone regeneration, emphasizing their function as 3D matrices that support osteoblast adhesion and new bone formation. It emphasizes chitosan’s biodegradable and biocompatible nature, making these scaffolds ideal for tissue engineering. The review also explores the combination of chitosan with HA and TCP to enhance osteoconductive and mechanical properties, thereby improving bone regeneration outcomes. Additionally, Tao et al [13] discussed chitosan’s capability to facilitate drug delivery, supporting the sustained release of growth factors, antibiotics, and bioactive molecules that aid in tissue repair.

Aranaz et al [14] provided a comprehensive review of chitosan’s technological applications, particularly in biomedical and drug delivery systems. They described how chitosan, because of its chemical versatility, can be modified to develop various drug delivery platforms for controlled and targeted release. Furthermore, the review discussed the mechanisms by which chitosan-based materials interact with bone cells, promoting osteogenesis and improving bone healing efficiency. The study also detailed how combining chitosan with nanoparticles, such as HA and TCP, further enhances its mechanical strength and bioactivity, making it a suitable candidate for bone tissue engineering and alveolar ridge preservation.

Recent randomized clinical trials have demonstrated that chitosan-based scaffolds are associated with reduced alveolar ridge resorption and improved radiographic bone density compared with natural healing following tooth extraction [12]. These findings support the biological rationale for incorporating CNPs into ridge preservation protocols and provide contextual evidence for the biological rationale of the present study.

### Limitations

The single-arm design and limited sample size restrict direct comparison with alternative socket preservation techniques or natural healing. Additionally, interindividual variability in socket morphology and biological healing responses, as well as the relatively short follow-up duration, may influence the observed outcomes. These factors will be considered when interpreting the study findings.

### Future Directions

This protocol may inform the design of future randomized controlled trials comparing CNP-based ridge preservation with conventional biomaterials. This protocol may inform larger randomized controlled trials comparing CNP combined with T-PRF to conventional ridge preservation materials. Future research may also focus on optimizing the nanoparticle concentration, evaluating long-term implant outcomes, and incorporating patient-reported outcome measures.

### Dissemination Plan

Study results, once available, will be disseminated through peer-reviewed journal publications, institutional presentations, and scientific conferences. Deidentified datasets and imaging outcomes may be shared in accordance with ethical guidelines and institutional policies, facilitating transparency and supporting future research collaborations.

## Abbreviations

BMSC: bone marrow mesenchymal stromal cell
BSP: bone sialoprotein
CBCT: cone beam computed tomography
CEJ: cementoenamel junction
CNP: chitosan nanoparticle
Col I: collagen type I
H&E: hematoxylin and eosin
HGF: human gingival fibroblast
IEC: institutional ethics committee
IL-1β: interleukin-1β
NLRP3: nucleotide-binding oligomerization domain–like receptor protein 3
PPP: platelet-poor plasma
PVA: polyvinyl alcohol
TCP: tricalcium phosphate
T-PRF: titanium-prepared platelet-rich fibrin

## Data Availability

The datasets generated or analyzed during this study will be available from the corresponding author upon reasonable request. Due to ethical restrictions and participant confidentiality requirements, raw clinical data cannot be publicly released. Aggregated results, cone beam computed tomography measurements, and anonymized histomorphometric images may be shared as multimedia appendices or through institutional data-sharing procedures upon approval from the ethics committee.

## References

[1] Ezoddini-Ardakani F, Navab Azam A, Yassaei S, Fatehi F, Rouhi G (2011). Effects of chitosan on dental bone repair. Health.

[2] Zhou H, Lee J (2011). Nanoscale hydroxyapatite particles for bone tissue engineering. Acta Biomater.

[3] Pippi R, Santoro M, Cafolla A (2017). The use of a chitosan-derived hemostatic agent for postextraction bleeding control in patients on antiplatelet treatment. J Oral Maxillofac Surg.

[4] Muzzarelli RA (2010). Chitins and chitosans as immunoadjuvants and non-allergenic drug carriers. Mar Drugs.

[5] Suh JK, Matthew HW (2000). Application of chitosan-based polysaccharide biomaterials in cartilage tissue engineering: a review. Biomaterials.

[6] Arnaud TM, de Barros Neto B, Diniz FB (2010). Chitosan effect on dental enamel de-remineralization: an in vitro evaluation. J Dent.

[7] Heimann RB (2020). Materials for Medical Application.

[8] Prabaharan M, Mano JF (2005). Chitosan-based particles as controlled drug delivery systems. Drug Deliv.

[9] Muzzarelli RA, Muzzarelli C, Heinze T (2005). Chitosan chemistry: relevance to the biomedical sciences. Polysaccharides I.

[10] Wang D, Li Q, Xiao C, Wang H, Dong S (2024). Nanoparticles in periodontitis therapy: a review of the current situation. Int J Nanomedicine.

[11] Yosouf K, Heshmeh O, Darwich K (2021). Alveolar ridge preservation utilizing composite (bioceramics/collagen) graft: a cone-beam computed tomography assessment in a randomized split-mouth controlled trial. J Biomed Sci Eng.

[12] Al-Madhagy G, Darwich K, Alghoraibi I, Al-Moraissi EA (2023). Radiographic evaluation of alveolar ridge preservation using a chitosan/polyvinyl alcohol nanofibrous matrix: a randomized clinical study. J Craniomaxillofac Surg.

[13] Tao F, Cheng Y, Shi X, Zheng H, Du Y, Xiang W, Deng H (2020). Applications of chitin and chitosan nanofibers in bone regenerative engineering. Carbohydr Polym.

[14] Aranaz I, Alcántara AR, Civera MC, Arias C, Elorza B, Heras Caballero A, Acosta N (2021). Chitosan: an overview of its properties and applications. Polymers (Basel).

